# We are not ready yet: limitations of state-of-the-art disease named entity recognizers

**DOI:** 10.1186/s13326-022-00280-6

**Published:** 2022-10-27

**Authors:** Lisa Kühnel, Juliane Fluck

**Affiliations:** 1 ZB MED - Information Centre for Life Sciences, Gleueler Str. 60, Cologne, Germany; 2 Graduate School DILS, Bielefeld Institute for Bioinformatics Infrastructure (BIBI), Faculty of Technology, Bielefeld University, Postfach 10 01 31, 33501 Bielefeld, Germany; 3 Institute of Geodesy and Geoinformation, Agricultural Faculty, University of Bonn, Nussallee 1, 53115 Bonn, Germany

**Keywords:** Text mining, bioNLP, BERT, Manual Curation

## Abstract

**Background:**

Intense research has been done in the area of biomedical natural language processing. Since the breakthrough of transfer learning-based methods, BERT models are used in a variety of biomedical and clinical applications. For the available data sets, these models show excellent results - partly exceeding the inter-annotator agreements. However, biomedical named entity recognition applied on COVID-19 preprints shows a performance drop compared to the results on test data. The question arises how well trained models are able to predict on completely new data, i.e. to generalize.

**Results:**

Based on the example of disease named entity recognition, we investigate the robustness of different machine learning-based methods - thereof transfer learning - and show that current state-of-the-art methods work well for a given training and the corresponding test set but experience a significant lack of generalization when applying to new data.

**Conclusions:**

We argue that there is a need for larger annotated data sets for training and testing. Therefore, we foresee the curation of further data sets and, moreover, the investigation of continual learning processes for machine learning-based models.

## Background

The amount of freely available, electronic data increased enormously in the biomedical field. Automatic information extraction methods have become indispensable and intense research has been done in the past. Whereas most text mining tasks were achieved with the help of rule-based systems in the beginning, mainly machine learning methods are used nowadays. The latter are strongly dependent on large amounts of curated data. However, manual curation is a complex and time consuming task, at least in the biomedical field, that needs to be done by domain experts. Hence, the availability of such high-quality data sets is strongly limited.

In the area of biomedical named entity recognition (NER), most data sets have been released for shared tasks and challenges open for the community. To name two examples, the national NLP clinical challenges (n2c2), formerly known as *i2b2 NLP Shared Tasks*, provide curated clinical data to researchers [1]; the organization *Critical Assessment of Information Extraction systems in Biology (BioCreAtivE)* organizes challenges for biological natural language processing (NLP) tasks and therefore also releases annotated data. In terms of disease entity recognition, to the best of our knowledge, two publicly available literature data sets exist that are commonly used: the National Center for Biotechnology Information (NCBI) Disease corpus [2] and the BioCreative V Chemical Disease Relation Task (BC5CDR) Disease corpus [3]. Both of the mentioned disease data sets follow the same annotation guidelines which are necessary to ensure consistency in annotations. These guidelines have been published together with the NCBI Disease corpus [4] and are also used for the more recent one (BC5CDR)[5]. Moreover, there are a few further data sets that contain disease named entities but were originally developed for related tasks, such as relation extraction. For example, Bagewadi *et al.* developed a corpus for the extraction of microRNA (miRNA) mentions and their relationships - thereof diseases [6]. The authors developed their own, simple annotation guidelines which state that disease terms are restricted to nouns, hence adjective terms are ignored. Moreover, the BioNLP13 Cancer Genetics (CG) data set is developed as event extraction corpus and contains annotated cancer-related disease terms [7]. Next to the existing corpora, we recently annotated 50 COVID-19 related articles with disease mentions [8].

Methodologically, the machine learning-based approaches applied to NLP have changed over time. First, methods like *support vector machines*, *hidden markov models* or *conditional random fields*, which all belong to the class of supervised algorithms, were often superior compared to rule based approaches. For those techniques, so-called features are needed to describe the input data. Examples of used features include general linguistic features (e.g. part-of-speech (POS) tags, stems), orthographic features (e.g. punctuation character, capitalized word) or dictionary look-up features. Later, so-called word embeddings - vector representations of words, usually learned over large collections of unlabeled data with the help of neural networks - replaced this feature engineering process [9]. These vectors are usually pre-trained with the objective to build a general language model, i.e. to predict the next word in a sequence. This principle can be understood as providing the neural network with prior knowledge about the nature of words and sentences - i.e. their semantics and syntax.

The aforementioned methods are all feature-based approaches: pre-trained representations (word embeddings) are included as features for a task-specific architecture [10]. More recently, so-called fine-tuning approaches have gained interest, which exploit a mechanism known as transfer learning. An already trained model is used as starting point to be trained on a new task. In case of NLP, the model is pre-trained on a general language understanding task and then fine-tuned on a specific NLP task like NER or relation extraction. With this shift in text mining methodologies, the complexity of the workflow is drastically reduced compared to rule- and feature-based approaches. Rule-based approaches require several pre-processing steps as for instance part-of-speech tagging, tokenization and sentence detection. Feature-based approaches rely on at least two different architectures, i.e. the creation of features and their inclusion into a (different) model. In contrast, fine-tuning based approaches only define one network architecture that is applicable to several different downstream tasks. The most popular network architecture is the bidirectional encoder representation from transformers (BERT) [10] that has been adapted to the biomedical area, called BioBERT [11], and shows state-of-the-art results for several different NLP tasks, thereof disease NER .

Based on the needs during the current COVID-19 pandemic, we set up the text mining-based semantic search engine preVIEW that automatically indexes preprints from several different sources [8, 12]. To recognize several entity classes (thereof diseases), we integrated publicly available ML-based models which show promising results of F1-scores above 85% for disease name recognition. Unfortunately, we realized a significant drop in performance when evaluated on a newly annotated COVID-19 preprint data set. With the implementation of an additional post-processing step^1^ that especially focuses on the recognition of COVID-19 related terms, their mapping to the new identifier and the removal of false positive entities that refer to the virus instead of the disease, we could achieve good results for this specific corpus [8].

These findings encouraged us to examine this performance reduction phenomenon in more detail - based on known data heavily used by the community: To the best of our knowledge, all recently developed systems for the recognition of diseases are trained and evaluated on either the NCBI or the BC5CDR corpus, on both of them separately or on the combination of these data sets. The question arises whether the models trained on these data sets are robust and applicable to real world applications.

In the current work, we investigate the similarities and differences of the two data sets and, in addition, compare them to a random PubMed data set in order to analyze the characteristics/bias of the different corpora. We also examine different NER algorithms – both transfer learning- and non transfer learning-based methods – and compare the performance of the algorithms trained on data set *A* and tested on the data set *B*. That is, we train a model explicitly on only one corpus and use the test sets of other corpora to obtain an independent evaluation of the quality of the model in terms of its ability to generalize. This is referred to as *cross evaluation* in the following. Additionally, we determine the performance of two of the algorithms trained on a merged corpus of both data sets (combined learning). Moreover, we evaluate the methods on the three above mentioned independent data sets: The first was developed for finding relationships between miRNAs and different biomedical entities, thereof diseases [6]. The corpus will be named miRNA-disease corpus in the following. Secondly, we evaluate the models on the BioNLP13-CG corpus which contains cancer-related disease terms [7]. Finally, we will use our own developed corpus which consists of 50 COVID-19 related articles that contain disease mentions (referred to as COVID Disease corpus in the following). Whereas, the latter relies on the annotation guidelines released with the NCBI corpus [4], the two other corpora come with their own annotation guidelines.

## Results

This section is subdivided into three different parts. First, we describe the results of the corpora comparison analyses. Afterwards, the results of the cross evaluations are described and finally we present the results of the combined learning approach.

### Semantic and linguistic comparison of data sets

**Fig. 1:**
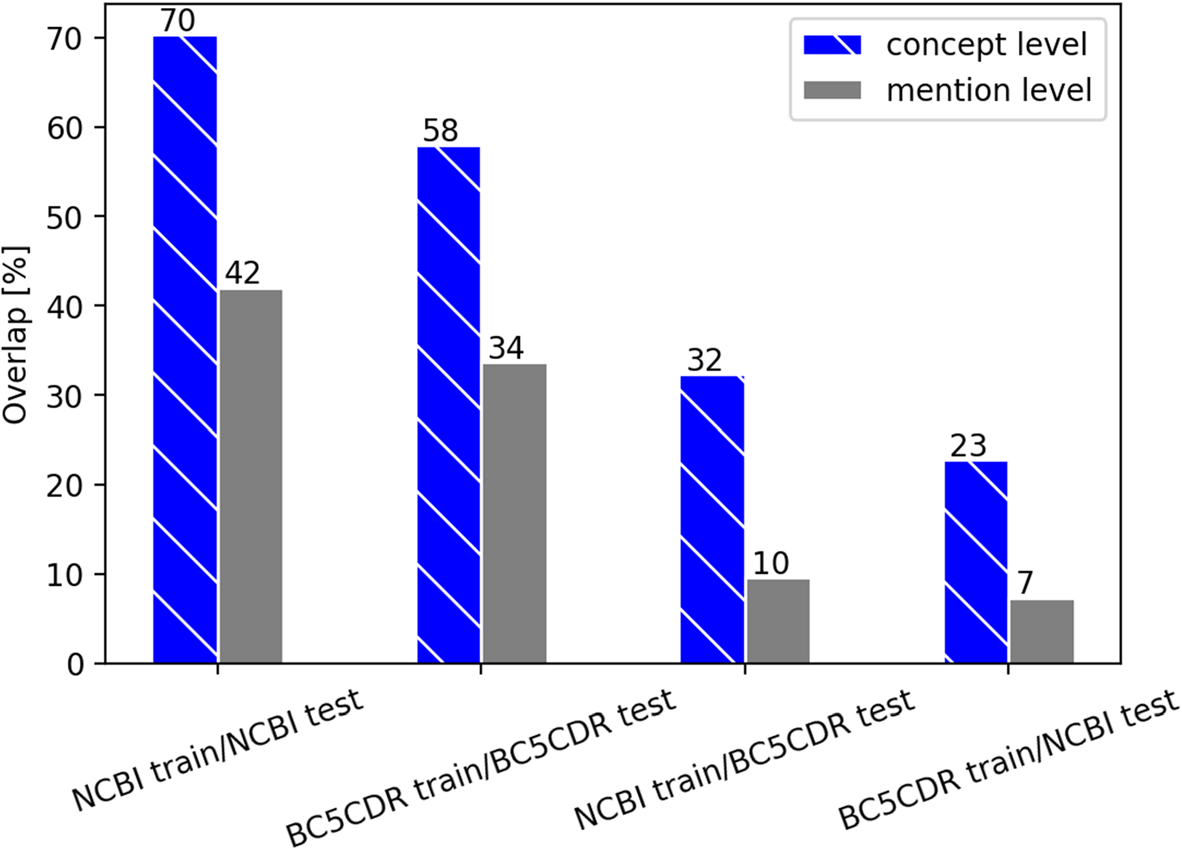
Semantic comparison of the NCBI and BC5CDR corpora on disease mention and concept level. The training sets are compared to their corresponding test sets. Additionally, the two different training sets are compared to the test sets of the respective other corpus

In a first step, we analyzed and compared the two main disease NER data sets (i.e. NCBI and BC5CDR data sets) in detail. We determined the overlap of both mentions and concepts between the training and the corresponding test set. The overlap between NCBI training and its test set reaches 70% on concept level, compared to an overlap of 60% between BC5CDR training and test set. Second, we determined the “cross-similarity”, i.e. the similarity of the training set of the NCBI corpus and the test set of the BC5CDR corpus and vice versa. The overlap between NCBI training set and BC5CDR test set only reaches 32% on the concept level and for the opposite case a value of 24% is reached. An overview of all results, also on the mention level, is given in Fig. 1. On the mention level, the overlap is lower within a corpus but we can also observe a drastic drop of cross similarity.

**Fig. 2:**
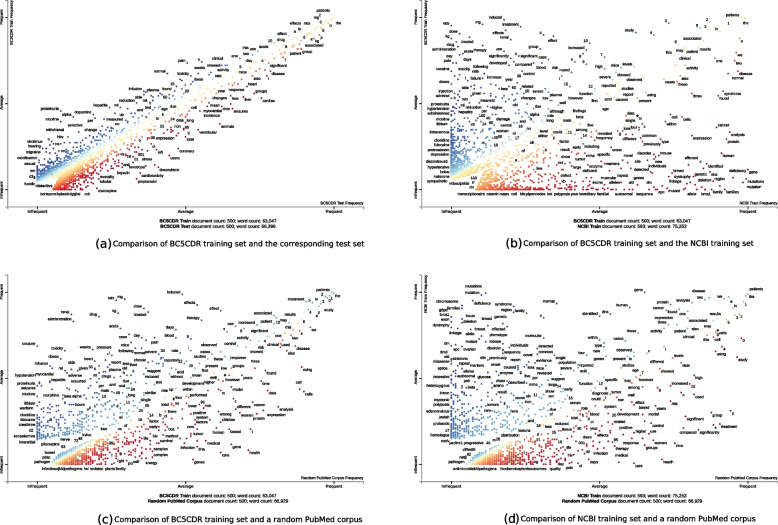
Comparison of the data sets with *scattertext*. On each axis, the frequency of a term is shown for the given documents. In Fig. 2a, the BC5CDR training set is compared to its given test set whereas in Fig. 2b, the BC5CDR training set is compared to the NCBI training set. In Figs. 2c and 2d, the BC5CDR training set and the NCBI training set are compared against a randomly chosen PubMed corpus of similar size

Moreover, we compared the linguistic variability of the different corpora using the visualization tool *scattertext* [13]. In Fig. 2a, we compared the BC5CDR training corpus to its corresponding test set. It shows a positive, linear relationship, indicating that the same words (or words with similar meaning) occur with similar frequency. In contrast, we do not see a relationship between the BC5CDR training set and the NCBI training set as the points are scattered throughout the whole plot (see Fig. 2b). This means, that terms that occur often in the BC5CDR training set occur rarely in the NCBI training set and vice versa. Finally, we compared both, the NCBI and the BC5CDR corpus, to the random PubMed corpus and received similar results (see Figs. 2c and 2d): in both cases also no linear trend can be seen but a widely distributed scatterplot. Whereas this might be expected for the BC5CDR corpus, as it only covers a specific domain (i.e. cardiovascular, neurological, renal and hepatic toxicity and their role in drug development), the NCBI corpus is intended to represent entire PubMed and the result is therefore rather unexpected.

### Cross Evaluation of NER models

**Fig. 3:**
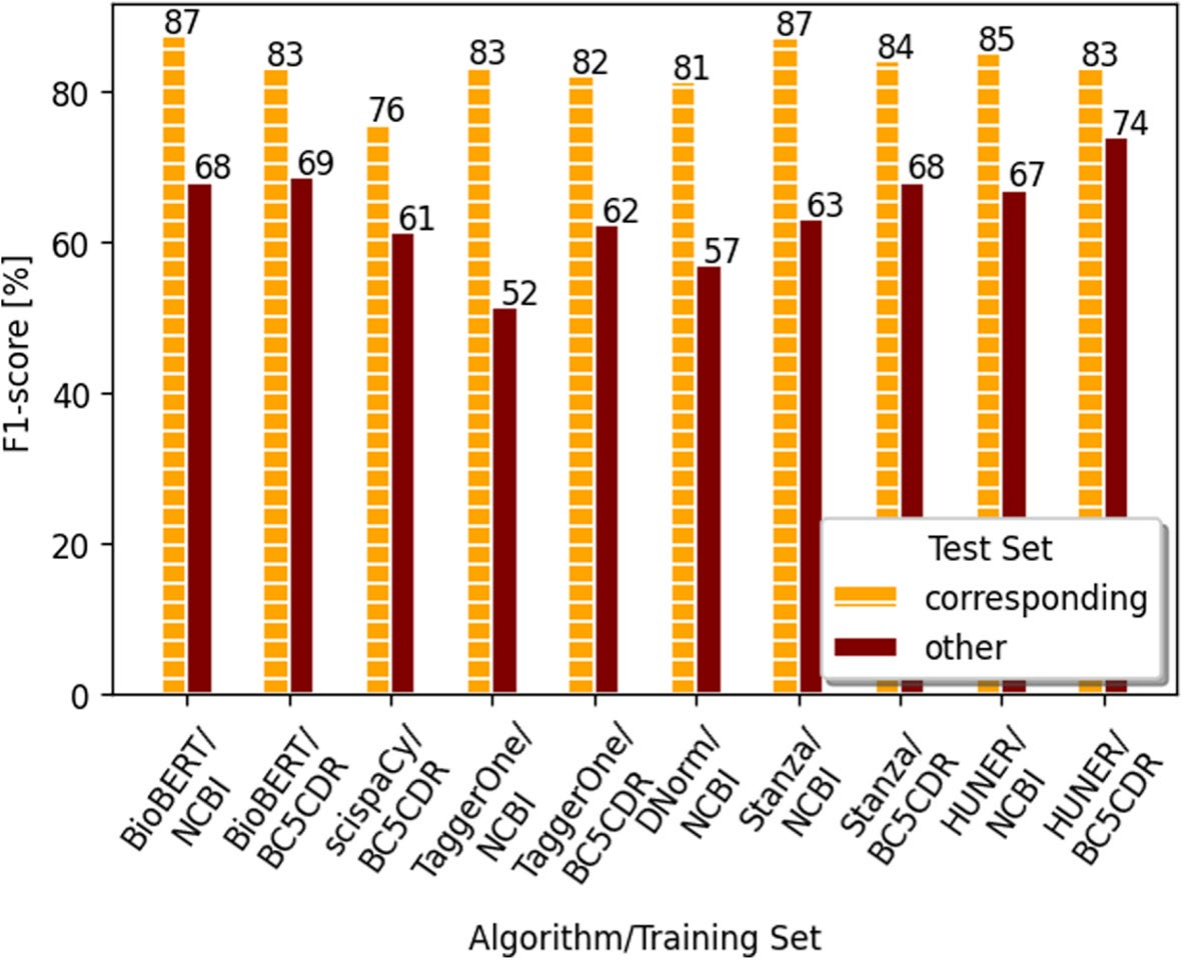
NER results for all tested ML algorithms. The F1-score is shown for the test set that belongs to the training set (corresponding test set) and to the test set of the respective other data set

In summary, we tested six different state-of-the-art machine learning algorithms, namely BioBERT, scispaCy, TaggerOne, DNorm, Stanza and HUNER. Whereas we trained BioBERT and HUNER in this study, we applied the other algorithms “as is”. An overview about the models can be seen in Table 1. The algorithms are further described in Section 5.2. All trained models are evaluated on both available test sets (NCBI and BC5CDR Disease). As can be seen in Fig. 3, the cross evaluation results in a significant drop for all used models. Whereas the BioBERT model trained on the NCBI training corpus achieves an F1-score of about 87% on the corresponding test set, it drops to 68% for the BC5CDR Disease test set. Similarly, the BioBERT model trained on the BC5CDR training set reaches an F1-score of 83% on the corresponding test set, the cross-evaluation, however, results in an F1-score of 69%. The highest difference is determined for the TaggerOne model trained on the NCBI training set. Whereas an F1-score of 83% for the corresponding test set is achieved, only 52% are reached for the BC5CDR test set. Vice versa, for the TaggerOne model trained on the BC5CDR corpus, we realize a 20% drop for the cross-evaluation. For trained DNorm, scispaCy, HUNER and Stanza models, the same trend has been determined. However, a slightly higher F1-score was determined for the HUNER model fine-tuned on the BC5CDR corpus: the F1-score amounts to 73.7% for the NCBI test set. This could be explained by the fact that the HUNER *disease-all* model that we used was, amongst others, pre-trained on the NCBI training corpus. Detailed results - including precision and recall - can be seen in Table 2. Interestingly, even though both precision and recall decrease, for all cross evaluations the drop of the recall is bigger than the drop of precision. For example, for BioBERT trained on the NCBI corpus, the recall drops by 22.34% whereas the precision drops by 18.52%. For TaggerOne trained on BC5CDR, the drop in precision amounts to 15.29%, and the difference in recall is 24.29%.

In addition, we evaluated the BioBERT models on three further related corpora that contain disease entities. As reference model, we use BioBERT trained on the respective training data set (if available). The results can be seen in Table 3. The BioBERT model trained on the miRNA-disease data set achieves an F1-score of approximately 80% on the corresponding test set. Both the NCBI and BC5CDR model perform only around 4% worse on the miRNA-disease test set. However, the BioBERT model trained on the NCBI corpus achieves only an F1-score of 61% on the BioNLP13-CG test set (in contrast to 86% when trained on the corresponding training set). An even worse F1-score can be seen when evaluating both the NCBI and BC5CDR model on the COVID-disease data set where F1-scores of 36% and 23% are achieved, respectively. This is mainly caused by the fact that the trained models are not able to predict newly evolved diseases, such as COVID-19.

**Table 1:** Overview of used training data sets for the respective algorithms

Training set			Algorithm			
	BioBERT	scispaCy	DNorm	TaggerOne	HUNER	Stanza
NCBI	$\checkmark$		$\checkmark$	$\checkmark$	$\checkmark$	$\checkmark$
BC5CDR	$\checkmark$	$\checkmark$		$\checkmark$	$\checkmark$	$\checkmark$
NCBI+BC5CDR	$\checkmark$			$\checkmark$		
miRNA-Disease	$\checkmark$					
BioNLP13-CG	$\checkmark$					

**Table 2:** Precision, recall and F1-score for both the corresponding test set and the respective other test set (i.e., cross evaluation)

Algorithm	Train set	Test set	Precision[%]	Recall[%]	F1-Score[%]
BioBERT	NCBI	NCBI	84.62	90.09	87.27
		BC5CDR	69.77	67.75	68.75
	BC5CDR	NCBI	73.63	63.19	68.01
		BC5CDR	82.07	85.39	83.07
TaggerOne	NCBI	NCBI	83.46	82.66	83.06
		BC5CDR	70.01	40.75	51.51
	BC5CDR	NCBI	68.30	56.38	61.77
		BC5CDR	83.59	80.67	82.11
scispaCy	BC5CDR	NCBI	65.65	57.49	61.30
		BC5CDR	76.20	75.22	75.71
DNorm	NCBI	NCBI	80.80	81.90	81.35
		BC5CDR	65.73	50.29	56.98
Stanza	NCBI	NCBI	86.65	88.54	87.58
		BC5CDR	70.24	57.78	63.40
	BC5CDR	NCBI	75.57	62.50	68.42
		BC5CDR	82.85	84.95	83.88
HUNER	NCBI	NCBI	83.82	86.35	85.07
		BC5CDR	70.20	64.92	67.46
	BC5CDR	NCBI	77.84	69.90	73.66
		BC5CDR	83.07	83.52	83.30

**Table 3:** Further cross evaluation results of BioBERT using related corpora

Train set	Test set	Precision[%]	Recall[%]	F1-Score[%]
MiRNA-disease	MiRNA-disease	78.63	80.60	79.60
BioNLP13-CG	BioNLP13-CG	86.01	86.47	86.24
NCBI	MiRNA-disease	71.96	81.53	76.45
BC5CDR	MiRNA-disease	72.74	80.59	76.47
NCBI	BioNLP-CG	50.14	79.09	61.37
BC5CDR	BioNLP-CG	48.60	75.19	59.05
NCBI	COVID Disease	46.24	29.66	36.13
BC5CDR	COVID Disease	30.64	18.28	22.89

### Learning on combined data set

Finally, we trained a BioBERT model on both NCBI and BC5CDR training data sets simultaneously and also evaluated this on both corresponding test data sets. Also for TaggerOne such a combined model is provided that we evaluated. As can be seen in Table 4, the results are similarly high for both test data sets. For BioBERT, the result on the NCBI test set is only 0.07% worse than the model only trained on NCBI; the result on the BC5CDR test set is even the same (see Fig. 3).

**Table 4:** Evaluation of models trained on combined NCBI and BC5CDR data set

Algorithm	Test set	Precision[%]	Recall[%]	F1-Score[%]
TaggerOne	NCBI	81.86	80.23	81.04
	BC5CDR	79.61	77.69	78.64
BioBERT	NCBI	85.19	88.74	86.93
	BC5CDR	82.07	85.21	83.61

## Discussion

In order to find relevant information in literature and hence to generate new knowledge, text mining methods have become indispensable because of the ever growing amount of electronic data. Therefore, a lot of research has been done in the area of bioNLP and current state-of-the-art algorithms show promising results on the available data sets. BERT is on everyone’s lips and used in a variety of biomedical and clinical applications [11, 14–16].

Because we integrated NER models into a semantic search engine and realized a drop in performance when evaluating an algorithm on a new data set, we started to question the robustness of current state-of-the-art methods. Therefore, in this work, we investigated the robustness of different machine learning-based algorithms on the task of disease named entity recognition. We chose this example because two different manually curated data sets are publicly available that are of similar size, basically follow the same annotation guidelines and are often used independently to develop and evaluate new methods. Assuming that the annotated disease corpora are large enough to train a model which generalizes and, in such a way is able to predict on new data, we evaluated the individually trained models on each other’s test set without further adjustment or training to test this hypothesis. Our analysis shows that none of the six tested algorithms performs nearly as good on cross evaluation as on the corresponding test set. Instead, we experience a significant drop in performance - on average 19% in terms of F1-score. To our mind, this can have the following two reasons: (1) the models can be overfitted towards the training data sets or (2) one such available corpus is simply not enough to learn this kind of complex biomedical NLP task. As we showed in our scatterplots, the content of the two used data sets strongly differ in content and wording and none of them represent the PubMed database (see Fig. 2). The specific content of a corpus is strongly dependent on the selection criteria, i.e. based on which strategy the abstracts were included. For example, the BC5CDR corpus was randomly selected from the CTD-Pfizer corpus [17] that contains 88,000 manually chosen and curated articles (abstracts) to investigate the potential involvement of pharmaceutical drugs in cardiovascular, neurological, renal and hepatic toxicity. Therefore, the BC5CDR corpus is focused on drugs and their role in toxicity.

To further investigate the models’ generalization ability, we used three additional data sets, originally developed for related tasks such as relation or event extraction. Whereas the drop in performance is relatively low for the miRNA-disease data set, we experience again a high drop for the BioNLP13-CG corpus. The lowest F1-score (amounting to 36% and 23% for the NCBI and BC5CDR models, respectively) is achieved for the COVID Disease data set that consists of relatively recent COVID-19 related articles.

As the BioBERT model trained on both the NCBI and the BC5CDR training sets reaches nearly the same results as each model trained on only one data set, the model is able to predict well on more variable test data if the training data set covers a similar variance. Therefore, the question arises when the model would be “ready” for real world applications - i.e. when we would have enough representative data. The model needs to be further tested on manually curated data that again covers a different area. However, such experiments are hampered by a lack of high-quality labeled data. Therefore, we foresee to set up a crowd sourcing-based approach in the near future and want to test the capabilities of transfer learning-based approaches for an active learning setup. Sequential fine-tuning of BioBERT models (i.e. re-training) experiences a mechanism known as *catastrophic forgetting* - the model forgets previously gathered knowledge and is biased towards the last data set [18]. Recently, so-called Adapter modules have been proposed that can be used for sequential learning of different tasks [19, 20]. However, it remains open how such methods perform on exactly the same – but highly variable and complex – task (i.e. disease NER in our case).

## Conclusions

Even though current transfer learning-based state-of-the-art methods for bioNLP show excellent results on the given training and corresponding test data, our analysis showed that those models are - against our expectations - not yet ready for real world applications because of a lack of generalization capabilities. Named entity recognition in the biomedical domain is much more complex than solving tasks on general domain knowledge, such as the recognition of persons or organizations. Moreover, a continual learning process is of great importance as the science progresses not only continuously but also rapidly. Therefore, in our future work, we foresee both the manual annotation of further data sets and the investigation of continual learning capabilities on this task in order to be able to solve real world cases.

## Materials and Methods

In the following, we first describe the used data sets. Afterwards, all six used algorithms are shortly described.

### Data sets

In the following section, we first describe the two main disease NER data sets (NCBI and BC5CDR) that follow the same annotation guidelines and are of comparable size. Thereafter, the three additional data sets are described. Thereof only one of the data sets follows the same annotation guidelines. The NCBI and BC5CDR corpora both consist of PubMed abstracts with manually curated disease annotations. The NCBI corpus with detailed annotation guidelines was released first. For the generation of the BC5CDR corpus, the previously published NCBI disease guidelines were re-used. The authors stated that “whenever possible, we will follow closely the guidelines of constructing NCBI disease corpus for annotating disease mentions” [21].

The NCBI Disease corpus was released by the National Center for Biotechnology Information (NCBI) and is “fully annotated at the mention and concept level to serve as a research resource for the biomedical natural language processing community” [2]. It contains 739 PubMed abstracts with a total of 6,892 disease mentions, annotated by a total of 14 annotators. Two annotators were given the same data so that a double-annotation could be performed. The inter-annotator agreement was determined by means of the F1-score (see Section 5.3) for each pair of annotators. The average F1-score amounts to 88% [2].

The BioCreative V Chemical Disease Relation (BC5CDR) was released by the organization BioCreative. The BC5CDR corpus contains 1,500 abstracts including disease and chemical annotations at mention level as well as their interactions (relations). In total, the data set contains 12,848 disease mentions [3]. For the present work, only the corpus containing disease mentions is used. Here, the inter-annotator agreement has been determined by means of the Jaccard distance. The Jaccard index divides the overlap of both sets (annotations) by the number in either set [22]. To determine the Jaccard distance, the index needs to be subtracted from one. The inter-annotator agreement amounts to 87.49% [3].

The NCBI training data set consists of 593 abstracts and the BC5CDR training data set consists of 500. In terms of unique mentions and concepts, they are also very similar. Whereas the NCBI training set contains 632 unique concepts, 649 can be found in the BC5CDR training set. In the test sets, huge differences can be found concerning the amount. The NCBI Disease test set only consists of 100 abstracts, the BC5CDR test set, however, consists of 500 abstracts as well. Therefore, the latter contains significantly more unique mentions and concepts. A detailed overview can be seen in Table 5.

**Table 5:** Statistics of used disease entity recognition data sets

	Data set	NCBI	BC5CDR	miRNA-disease	COVID Disease	BioNLP13-CG
Size (# Abstracts)		593	500	201	-	300
Unique mentions	training	1614	1445	461	-	349
Unique concepts		632	649	-	-	-
Size (# Abstracts)		100	500	-	-	200
Unique mentions	development	343	1343	-	-	154
Unique concepts		170	589	-	-	-
Size (# Abstracts)		100	500	100	50	100
Unique mentions	test	407	1432	224	68	260
Unique concepts		192	640	-	-	-

In our work, we analyze and compare these data sets on different levels: on mention level, on concept level and based on the whole corpus. For the latter, we apply the tool *scattertext* to visualize the linguistic variations [13]. In addition, we use the randomly generated PubMed corpus to perform a linguistic variation analysis between the annotated corpora and PubMed. This corpus was generated by randomly choosing 500 abstracts from all PubMed abstracts with a publication date between 1990 and 2021 (a total of 23,631,092 articles).

As three further, related data sets, we use the miRNA-disease corpus [6], the BioNLP13-CG corpus [7] and the COVID Disease corpus [12]. The miRNA-disease data set is split into training and test set. The training set consists of 200 abstracts, the test set consists of 100. The training set contains a total of 461 unique disease mentions, whereas the test set contains 224. In contrast to the NCBI and BC5CDR corpora, for this corpus, different, more simplified annotation guidelines were released that for example restrict the annotation to nouns. The BioNLP13-CG corpus consists of a total of 600 abstracts, split into training, development and test set. The test set contains 260 unique mentions. The COVID Disease data set is the smallest, consisting of 50 annotated abstracts. It has been developed as an independent test set for disease named entity models for COVID-19 related articles. Due its focus on COVID-19, it only contains 68 unique mentions.

### NER Algorithms

We investigated six different publicly available algorithms for disease named entity recognition in this work, that will be described in the following. Whereas we trained BioBERT and HUNER in this study, we applied the other algorithms “as is”. We provide an overview about the sources in the Availability Section. The applied algorithms will be described in the following.

BioBERT [11] is based on Bidirectional Encoder Representations from Transformers (BERT) [10]. As pre-trained model, we used *BioBERT-Base v1.0 (+ PubMed 200K + PMC 270K)* published by Lee *et al.* [11]. For fine-tuning, we used the library *Transformers* [23] and pytorch. In total, we trained five different models. First, we used the NCBI and BC5CDR training corpora and trained them both individually and on the combination on them. For the latter setting, the batches were shuffled randomly to avoid a higher influence of one data set over the other. The training parameters, investigated via cross-validation, can be seen in Table 6. Additionally, we used default parameters to train two further models on the miRNA-disease and BioNLP13-CG corpora (see also Table 6).

**Table 6:** Hyperparameters used for fine-tuning BioBERT

Corpus	Batch size	Learning rate	# of epochs
NCBI	32	5e-5	7
BC5CDR	32	3e-5	4
NCBI + BC5CDR	32	5e-5	4
BioNL13-CG	32	5e-5	3
miRNA-Disease	32	5e-5	3

scipaCy is based on the python library spaCy [24] that includes tools for text processing in several different languages. The text processing steps include for example sentence detection, tokenization, POS tagging or NER. Therefore, a convolutional neural network is used. scispaCy is trained on top of spacy for POS tagging, dependency parsing and NER using biomedical training data. The authors provide a model trained on the BC5CDR corpus to recognize diseases and chemicals. We used this model and filtered out the chemical annotations.

DNorm is a disease recognition and normalization tool [25]. It is a serial algorithm which uses first the entity recognition tool BANNER [26] based on conditional random fields (CRFs) which is followed by an abbreviation detection tool and a normalizer. Normalization is learned following a pairwise learning to rank approach. We apply the provided model trained on the NCBI Disease corpus.

TaggerOne is a joint named entity recognition and normalization model consisting of “a semi-Markov structured linear classifier, with a rich feature approach for NER and supervised semantic indexing for normalization” [27]. The authors provide three different models: one trained on the NCBI Disease corpus, one trained on the BC5CDR Disease corpus and one trained on both of them simultaneously.

Stanza is a python package that allows the building of machine learning-based NLP pipelines (including for example tokenizers or POS-tagger but also NER modules) for 70 different languages [28]. Zhang *et al.* published biomedical and clinical English model packages [29]. Optimized models for both the NCBI and the BC5CDR corpus exist and are used in this study.

HUNER, developed by Weber *et al.*, makes use of an LSTM-CRF-based architecture that is pre-trained in an semi-supervised manner and afterwards fine-tuned on a specific corpus/entity class [30]. To apply HUNER for our use-case, we downloaded the *disease-all* model and fine-tuned it on both the NCBI and the BC5CDR corpus, following the instructions of the authors (https://github.com/hu-ner/huner).

An overview about all available and/or trained models can be seen in Table 1.

### Evaluation Metrics

We determine precision, recall and F1-score to evaluate the models. The equations are given below, where FP stands for false positive, FN for false negative and TP for true positive. To ensure consistency, we use a publicly available evaluation script (CoNLLEval script) that has been released by the Conference on Computational Natural Language Learning (CoNLL) together with a shared task. The script is available under https://github.com/sighsmile/conlleval. This requires the input data to be in the “IOB”-format where each token is labeled as *B* for beginning, *I* for inside or *O* for outside. Evaluation is only done on entity, not on concept level and we only take exact matches into account.

1 $$\begin{aligned} precision = \frac{TP}{TP + FN} \end{aligned}$$

2 $$\begin{aligned} recall = \frac{TP}{TP + FP} \end{aligned}$$

3 $$\begin{aligned} F1-score = 2 * \frac{precision * recall}{precision + recall} \end{aligned}$$

## Data Availability

The NCBI Disease corpus and the BC5CDR corpus are both publicly available under https://www.ncbi.nlm.nih.gov/CBBresearch/Dogan/DISEASE/ and https://biocreative.bioinformatics.udel.edu/tasks/biocreative-v/track-3-cdr/, respectively. The miRNA-disease corpus has been downloaded from https://www.scai.fraunhofer.de/en/business-research-areas/bioinformatics/downloads/download-mirna-test-corpus.html. We retrieved the BioNLP13-CG corpus from https://github.com/cambridgeltl/MTL-Bioinformatics-2016/tree/master/data and the COVID Disease corpus is available under https://github.com/zbmed/preVIEW-COVID19/tree/main/data. The used algorithms are publicly available under: $\bullet$ BioBERT pre-trained models: https://github.com/naver/biobert-pretrained $\bullet$ Transformers library to fine-tune BioBERT: https://github.com/huggingface/transformers $\bullet$ scispaCy library: https://allenai.github.io/scispacy/ $\bullet$ DNorm: https://www.ncbi.nlm.nih.gov/research/bionlp/Tools/dnorm/ $\bullet$ TaggerOne: https://www.ncbi.nlm.nih.gov/research/bionlp/tools/taggerone/ $\bullet$ Stanza: https://stanfordnlp.github.io/stanza/biomed.html $\bullet$ HUNER: https://github.com/hu-ner/huner $\bullet$ Evaluation script: https://github.com/sighsmile/conlleval

## Abbreviations

BC5CDR: BioCreative V Chemical Disease Relation Task
BERT: Bidirectional Encoder Representations from Transformers
CoNLL: Conference on Computational Natural Language Learning
FN: False negative
FP: False positive
ML: Machine Learning
NCBI: National Center for Biotechnology Information
NER: Named Entity Recognition
NLP: National Language Processing
POS: Part-of-speech
TP: True positive

## Footnotes

1 The script is available under https://github.com/zbmed/preVIEW-COVID19/
